# Fabrication of Guided Tissue Regeneration Membrane Using Lignin-Mediated ZnO Nanoparticles in Biopolymer Matrix for Antimicrobial Activity

**DOI:** 10.3389/fchem.2022.837858

**Published:** 2022-04-19

**Authors:** Bushra Bilal, Rimsha Niazi, Sohail Nadeem, Muhammad Asim Farid, Muhammad Shahid Nazir, Toheed Akhter, Mohsin Javed, Ayesha Mohyuddin, Abdul Rauf, Zulfiqar Ali, Syed Ali Raza Naqvi, Nawshad Muhammad, Eslam B. Elkaeed, Hala A. Ibrahium, Nasser S. Awwad, Sadaf Ul Hassan

**Affiliations:** ^1^ Department of Chemistry, COMSATS University Islamabad, Lahore Campus, Lahore, Pakistan; ^2^ Department of Chemistry, School of Sciences, University of Management and Technology, Lahore Campus, Lahore, Pakistan; ^3^ Department of Chemistry, Division of Science and Technology, University of Education, Lahore, Pakistan; ^4^ Department of Chemical Engineering, COMSATS University Islamabad, Lahore Campus, Lahore, , Pakistan; ^5^ Department of Chemistry, Government College University, Faisalabad, Pakistan; ^6^ Department of Dental Materials, Institute of Basic Medical Sciences, Khyber Medical University, Peshawar, Pakistan; ^7^ Department of Pharmaceutical Sciences, College of Pharmacy, Almaarefa University, Riyadh, Saudi Arabia; ^8^ Biology Department, Faculty of Science, King Khalid University, Abha, Saudi Arabia; ^9^ Department of Semi Pilot Plant, Nuclear Materials Authority, Cairo, Egypt; ^10^ Chemistry Department, Faculty of Science, King Khalid University, Abha, Saudi Arabia

**Keywords:** ZnO nanoparticles, mucilage, lignin, GTR membrane, antimicrobial activity

## Abstract

Periodontal disease is a common complication, and conventional periodontal surgery can lead to severe bleeding. Different membranes have been used for periodontal treatment with limitations, such as improper biodegradation, poor mechanical property, and no effective hemostatic property. Guided tissue regeneration (GTR) membranes favoring periodontal regeneration were prepared to overcome these shortcomings. The mucilage of the chia seed was extracted and utilized to prepare the guided tissue regeneration (GTR) membrane. Lignin having antibacterial properties was used to synthesize lignin-mediated ZnO nanoparticles (∼Lignin@ZnO) followed by characterization with analytical techniques like Fourier-transform infrared spectroscopy (FTIR), UV–visible spectroscopy, and scanning electron microscope (SEM). To fabricate the GTR membrane, extracted mucilage, Lignin@ZnO, and polyvinyl alcohol (PVA) were mixed in different ratios to obtain a thin film. The fabricated GTR membrane was evaluated using a dynamic fatigue analyzer for mechanical properties. Appropriate degradation rates were approved by degradability analysis in water for different intervals of time. The fabricated GTR membrane showed excellent antibacterial properties against *Staphylococcus aureus* (*S. aureus*) and *Escherichia coli* (*E. coli*) bacterial species.

## Introduction

Guided tissue regeneration (GTR) techniques have been effectively used to treat periodontal problems and have supported the possibility of bone regeneration. GTR is a distinctive healing approach for periodontal infections (Caballé-Serrano et al., 2019). This technique uses the membranes as mechanical barriers to produce a gap around the flaws, allowing the formation of a new bone without the struggle for space by the nearby connective tissues. Membranes for GTR treatment must be biocompatible, have the appropriate degradation summary, have good physical and mechanical properties, and have enough continuous power (Shi et al., 2014; He et al., 2017; Khorshidi et al., 2018). GTR membranes should be permeable for cellular adaptation and adequate nutrient approval. Usually, the membranes are distributed into two classes: 1) non-resorbable and 2) bio-resorbable membranes. Non-resorbable membranes like expanded polytetrafluoroethylene (*e*-PTFE) should be detached after implantation through the surgical process.

On the other hand, bio-resorbable membranes involving collagen-based membranes are not essential to be removed since they reduce by period and do not need surgical removal (Caballé-Serrano et al., 2019; Ahmadi et al., 2020; Sadaf Ul et al., 2021). Moreover, non-biodegradable membranes made from titanium and polytetrafluoroethylene (PTFE) seem to be in great danger of postoperative problems as a second surgical process is necessary to recover implantations (Caballé-Serrano et al., 2019). So, biodegradable resources could be highly appropriate for the fabrication of the GTR membrane (Liu et al., 2020). Periodontal infections rise from constant inflammation, started by the accumulation of bacteria inside the dental tissue cells. Periodontitis development will have severe consequences like the fascination of alveolar bone tissues, the parting of epithelial tissues from the tooth, and the disruption of the periodontal ligament cells (PDL). Numerous biomaterials have been examined for the GTR application (Xue et al., 2014; Liang et al., 2020; Zhang et al., 2020). While choosing an ideal biomaterial for the GTR membranes, the subsequent needs must be measured, such as wound maintenance, space creation and protection, the safety of the fundamental blood clot, and the capability to eliminate undesirable tissues (Mota et al., 2012). Collagen has been one of the most common resources used to fabricate GTR membranes. However, this biomaterial is obtained from animal resources and has a connected danger of infection spread and related moral and ethnic issues, and causes immunogenic responses too.

Chitosan has drawbacks involving poor mechanical strength during its swelling, reducing its usage in various fields. Furthermore, its low mechanical power and the rapid resorption level of this biomaterial are a concern to several clinicians. In addition, by the enzymatic action of macrophages, polymorphonuclear leucocytes, and numerous microbes, fast collagen degradation can generate collagenase, which restricts membrane resistance to collapse. This result permits undesired cells to arrive at the wound place (Sela et al., 2003). Furthermore, the antibacterial action of the chitosan-based membrane is inferior at a neutral pH, restricting its use as an antibacterial (Hezma et al., 2019). Since collagen and chitosan-based membranes have presented such restricted properties, fresh and new resources with good properties are a requisite (Bottino et al., 2011). Natural polymers are sustainable and more biocompatible, and possess a higher biodegradable rate than synthetic polymers.

Moreover, natural polymers can display receptor-binding ligands to the cells. However, low mechanical strength restricts their application. For GTR applications, pure synthetic polymer or natural polymer scaffolds are generally insufficient. Pure synthetic polymers may have poor rigidity, hydrophobic character, or comparatively less bioactivity. On the other hand, the degradation results of the synthetic polymers are occasionally unfavorable for the freshly produced tissue. In contrast, the natural polymers may reduce so rapidly (biodegradable) to be utilized for the GTR. Therefore, an anticipated method is required that includes the usage of the combinations of synthetic and natural polymers in definite proportions to merge the benefits of both kinds (Abdelaziz et al., 2021).

Current reports show that using ZnO NPs also permits the development of bone growth, increases the osteoblast proliferation process, and prevents both Gram-negative and Gram-positive bacterial growth (Abdulkareem et al., 2015). For several years, Zn has also been utilized in dentistry as the main filler constituent of dental adhesives (Padovani et al., 2015). Lignin is an excellent applicant for the growth of new resources because of the phenolic and aliphatic hydroxyl functional groups in its structure and prevents the development of microbes, including *Staphylococcus aureus* and *Escherichia coli*. The lignin’s antimicrobial activity can decrease the danger of bacterial migration on the layer of the material. Mucilage, a natural extract from chia seeds, is unique (Muñoz et al., 2012). Mucilage has an excellent capacity for making films. There is growing attention to natural hydrocolloids for the production of decomposable films due to their non-toxic, low-cost, non-irritating quality, and other benefits (Beigomi et al., 2018). PVA is an extensively used, biocompatible, hydrophilic, water-soluble, and biodegradable synthetic polymer with more excellent mechanical applications appropriate for medical uses, including dental applications, bone regeneration, and wound healing (Zhou et al., 2020). The -OH groups in PVA can be a source of hydrogen bonding and help develop polymer composites (Khodiri et al., 2020).

**Scheme 1:**
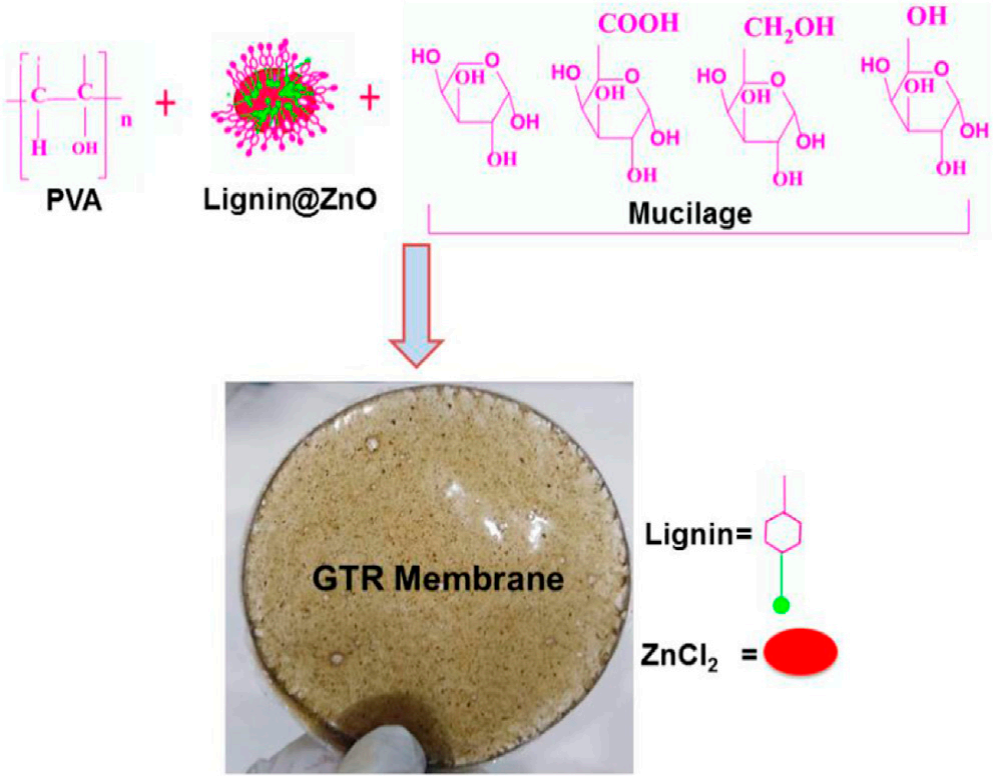
Schematic representation of the synthesis of GTR membrane.

In this study, we synthesized and introduced the Lignin@ZnO nanoparticles, used extracted mucilage from chia seeds to fabricate the GTR membrane, and used the PVA as a thickener and emulsion stabilizer (Scheme 1). Because microbial impurities are also the major reason for periodontal diseases, it is necessary to add materials or agents with antibacterial properties, prepare the infection-responsive GTR membrane, and increase its biological and mechanical strength. For this reason, Lignin@ZnO nanoparticles were added because of their broad-spectrum antibacterial properties.

## Experimental

### Materials

Zinc chloride (ZnCl_2_), sodium hydroxide (NaOH), Luria Bertani (LB) broth, and agar nutrients were obtained from Sigma Aldrich and used as received. PVA was purchased from Deajung. Chia seeds were purchased from the local market in Lahore, Pakistan. Ultra-filtered highly purified deionized water was utilized throughout the experimental procedure.

### Extraction of Lignin

Lignin was precipitated and attained by centrifugation and further purified by ethanol and ether. According to the previously reported method, lignin was extracted from bagasse (Joshi et al., 2019). First, 20 g bagasse was soaked in 200 ml HCl (0.1 N) solution for 24 h. Then it was filtered and washed with distilled water. Then bagasse was placed in 100 ml distilled water containing NaOH (3 mg) and 30% H_2_O_2_ (3 ml) and was irradiated with microwave in a microwave oven for 400 W for 30 min. This bagasse solution was filtered, and the filtrate was acidified by concentrated sulfuric acid to pH 4.

### Synthesis of Lignin@ZnO

The Lignin@ZnO nanoparticles were synthesized by using the probe sonication method. Solution A was prepared by adding 0.05 M zinc chloride (0.47 g) into 70 ml of deionized water. Sodium hydroxide (0.3 g) and lignin (0.6 g) were added to 30 ml of deionized water with continuous stirring until lignin was dissolved entirely (solution B). Afterward, solution B was added dropwise into solution A with further stirring for 15 min. The resulted solution was sonicated for 20 min at 60 amplitudes, using a pulse rate with a gap of 5 s. The synthesized sample was separated by centrifuge at 8000RPM for 10 min and dried in the oven at 80°C for 3 h.

### Extraction of Mucilage From Chia Seeds

The chia seeds were cleaned manually to eliminate dust particles. Chia seeds were added to the water with water and seeds in a ratio of 20:1 and continuously stirred at 450 RPM. To remove the mucilage layer from the surface of the seeds, the water–seed mixture was stirred by an overhead mixer using a four-blade propeller at 800 RPM for 2 h (Beigomi et al., 2018). Then mucilage was separated from seeds using cheesecloth (Supplementary Figure S1).

### Preparation of Membrane

PVA (1.6 g) was dissolved in mucilage (8 ml) with continuous stirring until a homogeneous mixture was formed. Three polymeric solutions were prepared in the PVA/mucilage solution by adding various ratios of Lignin@ZnO (1% (0.08 g), 2.5% (0.2 g), and 5% (0.4 g)). The resultant mixtures were stirred to improve the nanoparticles dispersions. Furthermore, 1% glycerol (0.08 ml) was added to each solution as a filler agent. The resulting solutions were stirred for a further half an hour to ensure that the solutions were completely homogeneous. The prepared solutions were poured into three petri dishes. The petri dishes were placed into the oven at 75°C for 3 h. After 3 h, the petri dishes were taken out of the oven, and three membranes of different compositions were prepared. The prepared membranes were evaluated for the antibacterial and mechanical study (Supplementary Figures S2, S3).

### Characterization

Chemical analysis (functional group identification) of the reaction mixture was performed using Fourier transform infrared spectroscopy (FTIR, Thermo Scientific Nicolet 6700) over a wavelength range from 400 to 4000 cm^−1^ at a resolution 4 cm^−1^. The optical properties of the synthesized Lignin@ZnO were characterized using UV–visible spectroscopy. UV spectra of the reaction mixture were recorded at (LAMBDA 25) spectrophotometer in the wavelength range of 200–800 nm, whereas resuspending ZnO NPs monitored the stability of ZnO NPs in distilled water. These aqueous suspensions were sonicated at room temperature (25 min) for the uniform dispersion of ZnO NPs. The surface and cross-sectional morphologies of the fabricated membranes were studied by scanning electron microscopy (SEM, TESCANV LMU). The fabricated novel membranes were observed under an optical microscope (Optika B-600 MET) to evaluate the membrane’s morphology and overall fiber structure. The mechanical properties (i.e., tensile strength, Young’s modulus, and elongation at break) of all the fabricated membranes were evaluated by using a universal testing machine (electrodynamics fatigue testing machine, Walter + bai AG, Switzerland). Rectangular membranes (30 mm × 5 mm) were tested. The crosshead speed was set at 5 mm/min. The tensile modulus was calculated from the slope of the initial linear part of the curve (Castro et al., 2018). A swelling test determined the swelling index of the membrane. The water content of the GTR membrane was determined by swelling the membrane in pH 7.4 of phosphate-buffered saline (PBS) at room temperature. The dry weight of the membranes was measured to estimate the amount of antibiotic solution that the membrane could absorb. After measuring the dry weight, the membranes were immersed in PBS for different time intervals (30 min, 1, 2, 4, and 24 h) to confirm the perfect swelling.

$$\mathrm{Water}\;\mathrm{intake}\left(\%\right)=\frac{W_{w}-W_{i}}{W_{i}}\times 100,$$ (1)

where W_*i*_ is the initial weight of the sample and W_*w*_ is the weight of a sample after dipping in PBS (Lian et al., 2019). The *in vitro* antibacterial activity of the fabricated membrane-synthesized Lignin@ZnO and commercial zinc oxide powder was examined against *Staphylococcus aureus* (*S*. *aureus*, ATCC 6538 a representative Gram-positive bacteria) and *Escherichia coli* (*E coli*, ATCC 8739 a representative Gram-negative bacteria) through the detection of the inhibition zone using the disc diffusion method. After that, 0.32 g of nutrient broth was taken in two different flasks with 25 ml of water in each flask, and it was then autoclaved at 121 °C for 21 min. It was cooled, and in one flask, Gram-positive bacteria were added, and in another flask, Gram-negative bacteria were added. These flasks were kept for 24 h in a shaker. Then in another flask, 200 ml water was taken and 2 g of nutrient broth and 4 g agar were added to it. It was heated and stirred until the water boiled. Then this mixture was poured into a petri dish and was cooled, and the flask placed in the incubator was taken out from the incubator after 24 h of incubation. Bacterial culture was carried out using the incubated bacteria, and nutrient agar plates were swabbed with bacterial strain broth to form a lawn growth of *E*. *coli* and *S*. *aureus*. Small pieces of the prepared membrane were placed on the petri plates. The plate was then placed in an incubator at 37 °C for 24 h (Lian et al., 2019).

Amoxicillin and tetracycline disks were used as standard antibiotic discs. Tetracyclines are a collection of broad-spectrum antibiotics that can prevent protein synthesis by binding to the ribosomal subunit in the mRNA translation complex (Qiu et al., 2020). Amoxicillin is used to treat a large diversity of bacterial contaminations. This medication is a penicillin-type antibiotic. It works by preventing bacterial growth. This antibiotic treats only bacterial infections. Amoxicillin is used in dentistry to treat dental alveolar abscesses, endodontic infections, and advanced forms of periodontal diseases. So, elements that promote an increase in amoxicillin resistance in the oral microbiota are of concern to both medical and dental practitioners (Zhao et al., 2021). After 24 h, plates were taken out from the incubator, and the diameter of the zone of inhibitions around the antibiotic disc was measured in millimeters. The experiments were performed in triplicate. Results showed that both synthesized Lignin@ZnO and fabricated novel membranes presented prominent bactericidal activity against both bacterial strains.

### Morphology Investigation of Bacteria

The cultures of bacteria were centrifuged for 2 min at 6,000 rpm, washed three times with phosphate-buffered saline (PBS, 7.4), and mixed with 2.5% glutaraldehyde solution for 4 h. The cells were dehydrated with consecutive treatment of 30, 50, 70, 80, 90, and 100% ethanol for 15 min, respectively. The dehydrated cells were coated with gold by sputtering under vacuum and imaged using a FE-SEM. Using the FE-SEM, the morphological changes of bacterial cells were observed after treating the samples for 24 h.

## Results and Discussion

### FTIR Analysis

**FIGURE 1:**
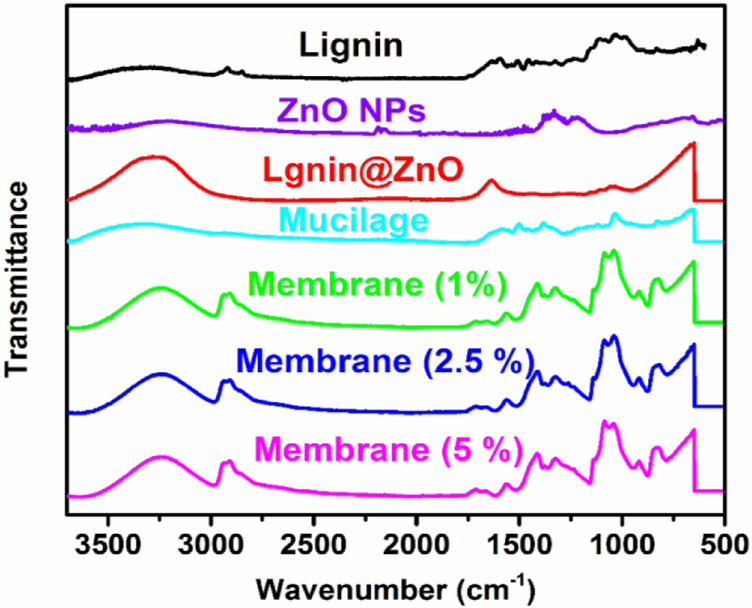
FTIR spectra of lignin, ZnO NPs, Lignin@ZnO, mucilage, 1, 2.5, and 5% Lignin@ZnO-GTR membranes.

FTIR spectra was used to identify the presence of different functional groups in the tested samples. FTIR spectra of ZnO, mucilage, Lignin@ZnO, and 1, 2.5, and 5% Lignin@ZnO-GTR membranes is shown in Figure 1 while all the frequencies exhibited by the different functional groups are compiled in Table 1. The pure ZnO spectrum peaks at 530 cm^−1^, which shows the *ν*-Zn-O stretching frequency (Jose et al., 2020). Mucilage shows various peaks at 3,340 cm^−1^, 1,045 cm^−1^, 1,607 cm^−1^, and 1,508 cm^−1^ for *ν*-OH (broad), *ν*-C-O-C of glycosidic linkage imide-1 in chia seeds mucilage, and *ν*-COO for uronic acids, respectively (Darwish et al., 2018). Spectrum for Lignin@ZnO shows peaks at 3,270 cm^−1^ (broad, *ν*-OH stretching), 1037 cm^−1^ (*ν*-C-O-C stretching) for phenol units, and 1,639 cm^−1^ (*ν*-C-H stretching aliphatic) (Jose et al., 2020). The peaks at 1,080 cm^−1^ and 1,180 cm ^−1^ are characterized as C-O-C stretching. The FTIR spectrum for Lignin@ZnO exhibits almost all peaks of pure lignin, which indicates that mediation of ZnO into lignin is successfully done. Different membranes are formed by adding mucilage and different loading ratios of Lignin@ZnO, which shows other peaks corresponding to mucilage and lignin. The FTIR spectrum of membranes shows a peak at 3,251 cm^−1^ (ν-OH stretching), but the peak value is lower than the lignin and mucilage -OH peak, which confirms the presence of hydrogen bonding between different components in the membrane (Pulit-Prociak et al., 2020).

**TABLE 1:** FTIR data of ZnO, Lignin@ZnO, mucilage, 1, 2.5, and 5% Lignin@ZnO-GTR membranes.

ZnO	Lignin	Lignin@ZnO	Mucilage	Membranes			Assignments
				1%	2.5%	5%	
-	-	3,270	3,344	3,251	3,239	3,245	*ν*(OH stretching) from carbohydrate and lignin
-	-	-	-	2,928	2,923	2,923	*ν*(C-H aromatic) from phenolic lignin
-	-	-	-	1707	1707	1719	*ν*(C=O stretching) from carbohydrate
-	-	1,639	1,601	1,564	1,551	1,558	*ν*(C-H aliphatic stretching) from mucilage
-	1,457	1,639	-	1,558	1,570	1,558	*ν*(C=C aromatic stretching) from lignin
-	-	-	1,508	1,409	1,415	1,421	*ν*(-COO carboxyl group) from mucilage and uronic acid
-	1,515	-	-	1,409	1,415	1,415	(*ν*-CH_3_ stretching) from lignin phenolic compounds
-	-	1,130	1,124	1,130	1,130	1,136	*ν* (C-O bending) from pyranose
-	-	1,043	1,037	1,043	1,037	1,031	*ν*(C-O-C) stretch and *ν*(C-O-H) bending
-	-	-	1,037	1,056	1,043	1,074	*ν* (C-O-C stretching) from glycosidic linkage
500–600	-	525	-	528	527	529	Stretching of ZnO NPs

A fragile band around 1,500 cm^−1^ is the characteristic band of the lignin (Câmara et al., 2020). A peak around 1,457 cm^−1^ is a deformation of lignin CH_2_, and CH_3_ is stated to be stretching the C=O and C=C lignin aromatic rings (Lun et al., 2017). Membranes also show stretching frequency at 2,928 cm^−1^ for aromatic *ν*-C-H bonds, indicating the presence of phenolic units from lignin. A peak at 1,037 cm^−1^ showed the C-O-C glycosidic linkage from mucilage polysaccharides. In membranes, band at 1,419 cm^−1^ indicates the CH_2_ bending, the peak at 1,165 cm ^−1^ shows the stretching of C–O, the band at 1,080 cm^−1^ to 1090 cm^−1^ represents the bending of the OH group, and the peak at 843 cm^−1^ carbon–carbon stretching. Furthermore, the peak at 1,409 cm^−1^ (for *ν*-COO carboxylic group) from uronic acids of polysaccharide of mucilage, 1,558 cm^−1^ (for *ν*-C=C stretch) from phenolic units, 1,564 cm^−1^ (aliphatic *ν*-C-H stretching) from lignin and mucilage, and 500–600 cm^−1^ from ZnO further confirm the formation of the GTR membrane (Figure 1 and Table 1).

### UV–Visible Analysis

**FIGURE 2:**
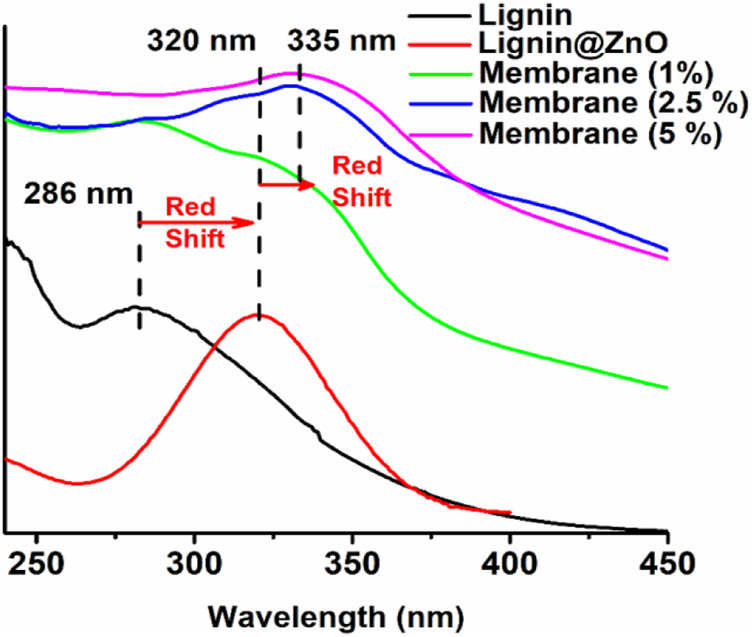
The UV–visible spectrum of lignin, Lignin@ZnO, 1, 2.5, and 5% Lignin@ZnO-GTR membranes.

The UV–visible spectra of lignin, Lignin@ZnO, 1, 2.5, and 5% membranes are shown in Figure 2. The peak at 286 nm is attributed to extended conjugation with different substituents on the cyclic benzene ring of lignin (Joshi et al., 2019). The peak at 320 nm is associated with lignin-mediated ZnO nanoparticles (Lignin@ZnO). The absorption peak shift toward the higher wavelength (redshift) is observed twice during this whole procedure: first, during the complex formation of Lignin@ZnO and second, when the GTR membrane was formed by adding the mucilage and PVA into the Lignin@ZnO with different loading ratios such as 1%, 2.5%, and 5% (wt./wt.). The peak intensities are increased as we increase the loading ratios of Lignin@ZnO. The broad peaks are observed at ∼335 nm for differently loaded membranes (1%, 2.5%, and 5%). This redshift phenomenon confirmed the Lignin@ZnO and GTR membrane formation in the first and second steps, respectively (Patra et al., 2014; Joshi et al., 2019; Jose et al., 2020).

### XRD Results

**FIGURE. 3:**
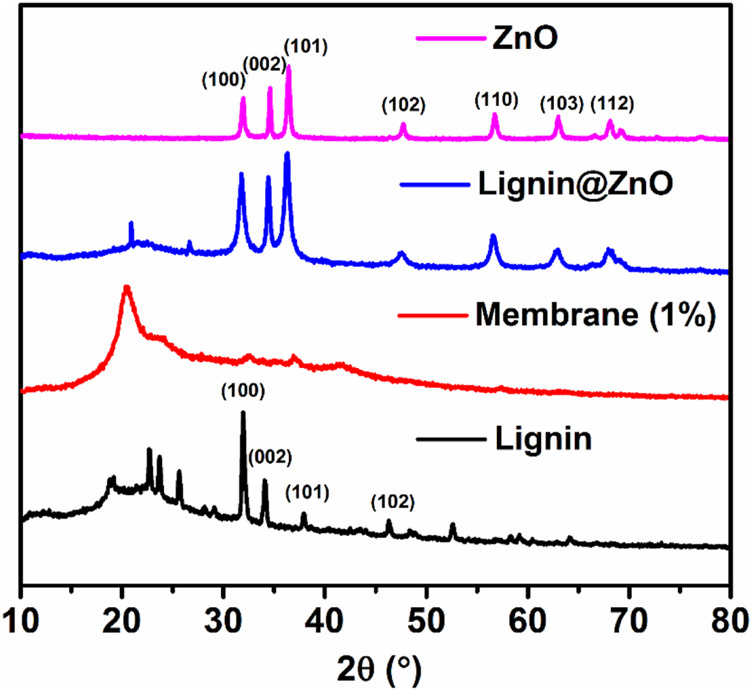
XRD spectra of synthesized ZnO NPs, Lignin@ZnO, Lignin, and fabricated membrane (1%).

The crystalline structure, crystal orientation, phase purity, and the crystallite size of the synthesized ZnO NPs, Lignin@ZnO, pure lignin, and fabricated GTR membrane were characterized by XRD analysis. XRD diffractogram of ZnO NPs and Lignin@ZnO displayed characteristic peaks at 31.87°, 34.54°, 36.46°, 47.67°, 56.85°, 62.84°, and 68.19°, which can be attributed to 100, 002, 101, 102, 110, 103, and 112 reflection planes of a hexagonal wurtzite structure of ZnO NPs (Figure 3).

Similarly, a diffractogram of lignin displays the diffraction peaks at 31.87°, 34.16°, 37.99°, 46.27°, and 52.51°, which also agree with 100, 002, 101, 102, 110, 103, and 112 reflection planes. Previously, many studies have reported the hexagonal wurtzite structure of ZnO NPs. The vigorous intensity and narrow width of diffraction peaks of both types of synthesized ZnO NPs revealed that the resulting nanoparticles were highly crystalline (Abbasi et al., 2017). Lignin@ZnO showed an intense peak, which belonged to the (100) plane; it was higher than that observed for the (100) plane. As no characteristic peak for impurity originated, the synthesized ZnO nanoparticles can be considered to have excellent crystalline nature. The main change was detected in the peak widths with the similarity in the unit cell parameters of both forms of synthesized ZnO nanoparticles with lignin and without lignin. The broadening of peaks in XRD was a consequence of a decrease in crystallite size. In the XRD pattern of the GTR membrane, the diffraction peak situated at 20.51° is allocated to a semicrystalline structure of the GTR membrane (Joshi et al., 2019).

### SEM Study

**FIGURE 4:**
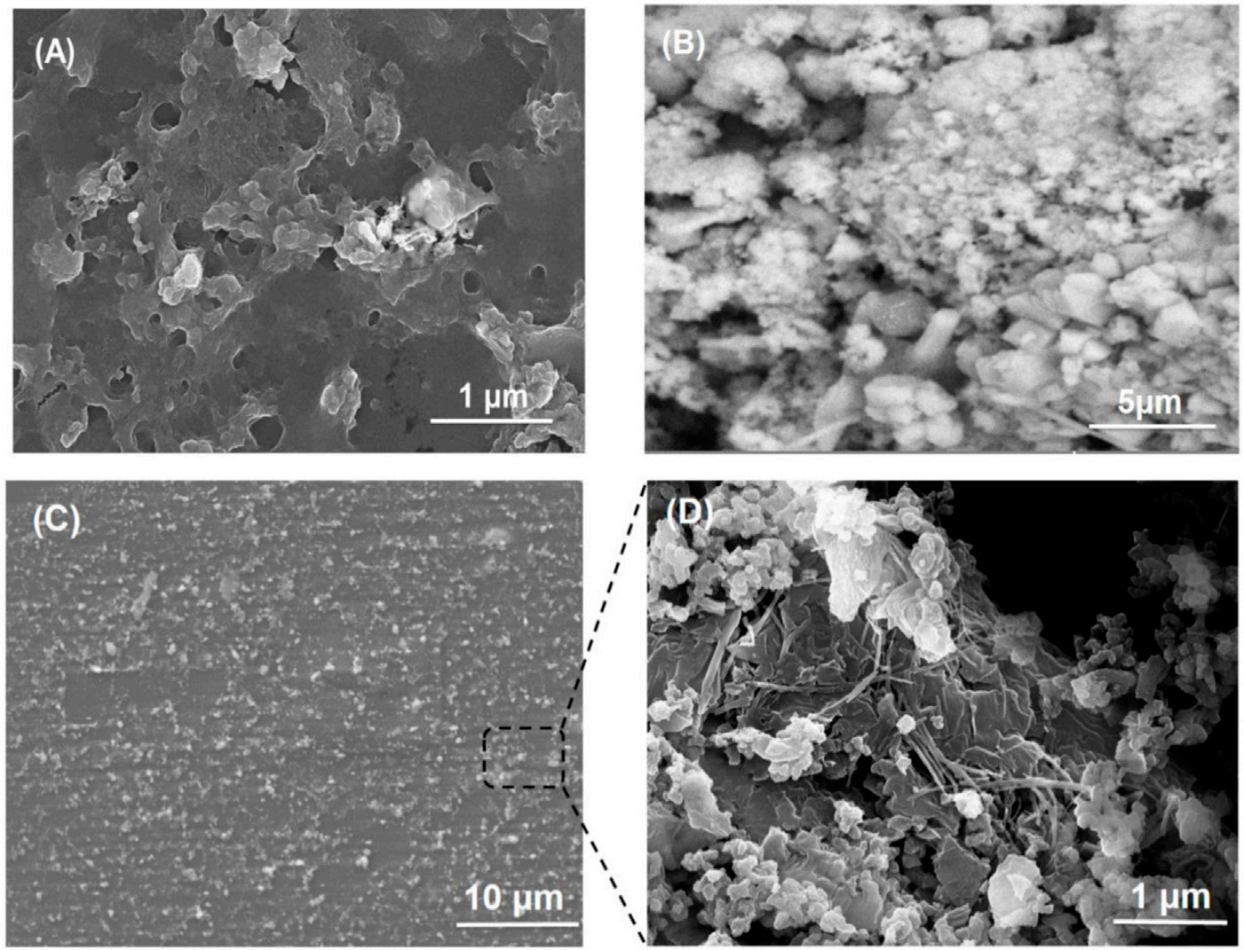
SEM images of **(A)** PVA/mucilage, **(B)** Lignin@ZnO, and **(C)** Lignin@ZnO-GTR membrane, and **(D)** zoom image of the cross section from (C).

SEM images of PVA/mucilage, Lignin@ZnO, and Lignin@ZnO-GTR membrane (containing 1% @Lignin@ZnO) are shown in Figure 4. Figure 4A shows an SEM micrograph of chia seed mucilage with PVA. When PVA is added with chia seed mucilage, then there will be smooth morphology like a polymer structure inside (Dehghani et al., 2020); on the upper surface, agglomeration is shown due to evaporation of water from the surface that would lead toward the partial insolubility of mucilage and its accumulation (Salazar Vega et al., 2020). In this study, attained ZnO NPs seemed irregular in shape, and the size of the particles is in the nano range, mostly less than 50 nm. However, the particles are agglomerated, as shown in Figure 4B. The obtained results clearly illustrate that the Lignin@ ZnO was well prepared without impurities. But in the membrane, Lignin@ZnO particles dispersed throughout the membrane because of biopolymer addition and cavities provided by those polymers. EDX studies of ZnO NPs and membranes are shown in Supplementary Figures S4, S5.

### Morphological Analysis

Figure 4 shows the microscopic evaluation of the membranes in which randomly interconnected structures, smooth morphology, and distributions of Lignin@ZnO are observed. As stated previously, morphological examinations of membranes revealed that Lignin@ZnO percentage increased, and fibers became rougher in membranes (Münchow et al., 2015). The water drop could not penetrate wild and smooth fibers. The observed difference could be a consequence of the change in the surface properties of membranes like roughness (Türkkan et al., 2017). However, it could be found that the morphology of fibers, including diameter, porosity, and surface structure, was influenced by many processing parameters such as viscosity of suspension determined by polymer concentration and additives (Ahmadi et al., 2020).

When biopolymers (PVA/mucilage) solutions are mixed with Lignin@ZnO, they exhibit the electrostatic force of interactions (i.e., hydrogen bonding) between PVA, mucilage, and Lignin@ZnO. During the hydrogen bonding formation, the interaction of polymers with each other would not be fine but random-like structure because of the extraction procedure. When the extraction is applied, it causes disturbance in the orientation of the polymer matrix. Figure 4 membrane image shows some cracks presumably caused by air bubbles incorporated during membranes formation (Figure 4B). Moreover, some particles with thread forms (increased as Lignin@ZnO concentration increased) were observed, which can be attributed to the mucilage structure (Beigomi et al., 2018).

### Mechanical Properties

**FIGURE. 5:**
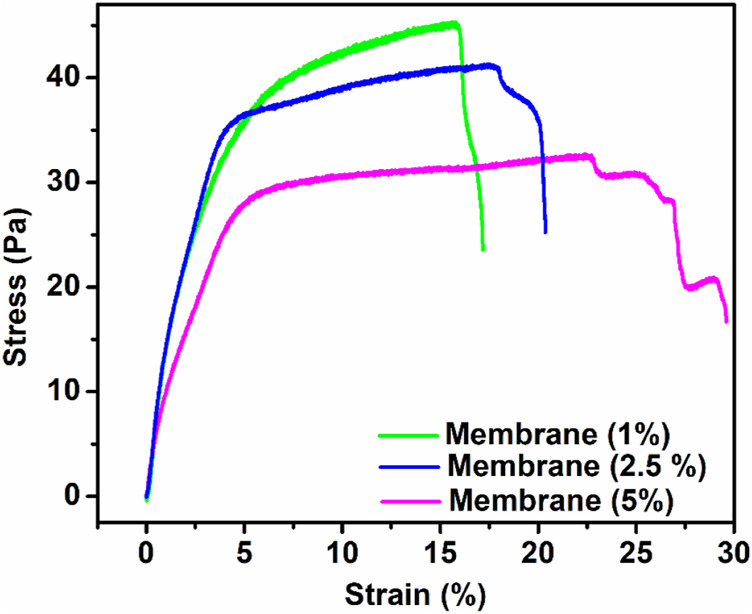
Strain (percentage) and tensile strength of the synthesized membrane having a different ratio of ZnO NPs.

First, prepared membranes were cut into 5-mm-width and 35-mm-long strips. The load–deformation curves at a speed of 5 mm/min were recorded (He et al., 2019) to obtain the stress–strain curves of the membranes. The mechanical performance (e.g., tensile strength, Young’s modulus, and strain) of the synthesized membranes is presented in Table 2. According to previous results, the mechanical properties of the polymer matrix composites filled with nanoparticles could be influenced by fixed or variable parameters such as the chemical composition of matrix and filler and interaction between them, membrane architecture, morphology, size, and dispersion nanoparticles. Among the aforementioned parameters, distribution of Lignin@ZnO, fiber diameter, and interaction between Lignin@ZnO and polymer chains play essential roles in the final mechanical properties of the membrane (Ahmadi et al., 2020). The addition of the Lignin@ZnO decreased the mechanical characteristics; however, strain increased, which was improved with an increased percentage of Lignin@ ZnO, as shown in Figure 5 (Münchow et al., 2015).

**TABLE 2:** E modulus, strain (percentage), and tensile strength of the synthesized 1, 2.5, and 5% Lignin@ZnO-GTR membranes.

Sample	E-modulus (GPa)	Tensile strength (Pa)	Strain percentage (%)
Lignin@ZnO-GTR membranes (1%)	0.12	22.66	66.43
Lignin@ZnO-GTR membranes (2.5%)	0.07	18.73	71.42
Lignin@ZnO-GTR membranes (5%)	0.19	14.36	91.59

The tensile strength of the membrane having Lignin@ZnO (5 wt%) was lower than the other membrane having 2.5 and 1 Lignin@ ZnO wt%. It may be attributed to the fact that during solution casting, the Lignin@ZnO particles dispersed in the polymer matrix decreased the physical cross-link strength between the polymer blend chains and Lignin@ZnO due to aggregation nano-filler in a polymer matrix; as a result, fractures quickly developed. The increase in toughness value with an increase in the content of Lignin@ZnO has been ascribed to the rise in the polymeric matrix local density. This result reveals that the dispersion of the Lignin@ZnO in composites plays a significant role in determining the elasticity modulus, tensile strength, and toughness (Hezma et al., 2019). Enhancing the ratio of the Lignin@ZnO causes the disposition of the accumulations. The occurrence of these bunches reduced the actual weight move from the polymer medium to the filler by decreasing the surface capacity in association with NPs and causing the stress ratio, causing the lower stress transmission at the boundary and eventually the mechanical characteristics (Figure 5; Table 2) (Makaremi et al., 2016).

### Swelling Properties

**FIGURE 6:**
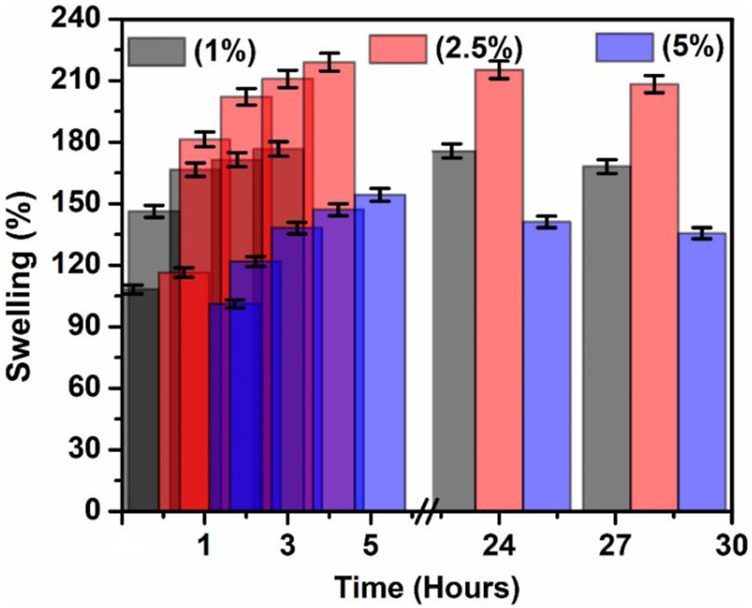
Swelling property of the 1, 2.5, and 5% Lignin@ZnO-GTR membranes.

The detection of water absorption properties is essential in studies on barrier membranes. Higher water absorption performance results in better nutritional uptake ability and exudate absorption by a GTR membrane, which will promote cell growth and the absorption of inflammatory exudates by the GTR membrane. Different types of cross-linked hydrogel swelling studies have been conducted using this procedure (Türkkan et al., 2017). Then there is a slight decrease in swelling capacity for all samples until equilibrium after 24 h was reached (Kowalski et al., 2019). The degree of swelling also decreases with the increasing concentration of Lignin@ZnO (He et al., 2017). Based on the literature, the appropriate penetration, uniform distribution, and effective size of zinc oxide nanoparticles may decrease the hygroscopicity (Soltani et al., 2013). The swelling data show that the membrane having 1% Lignin@ZnO has the highest swelling ratio and that of the 5% Lignin@ZnO membrane was the smallest, as shown in Figure 6. Analyzing the water absorption results, the maximum amount of water absorption after 30 min can be observed. The result indicates that an increased concentration of Lignin@ZnO reduced the actual water absorption performance of the GTR membrane. The swelling degree of each of the membranes is presented in Table 3.

**TABLE 3:** Membrane swelling degrees of various types of fabricated membranes.

Time	Swelling ratio of Lignin@ZnO-GTR membrane (1 %)	Swelling ratio of Lignin@ZnO-GTR membrane (2.5 %)	Swelling ratio of Lignin@ZnO-GTR membrane (5 %)
30 min	108.2	116.4	101.1
Hour	146.3	181.3	121.8
2 h	166.6	202.1	138.1
3 h	171.4	210.9	147.1
4 h	176.6	219.0	154.3
24 h	175.7	215.3	141.1
28 h	168.1	208.3	135.5

### Antibacterial Activity

**FIGURE 7:**
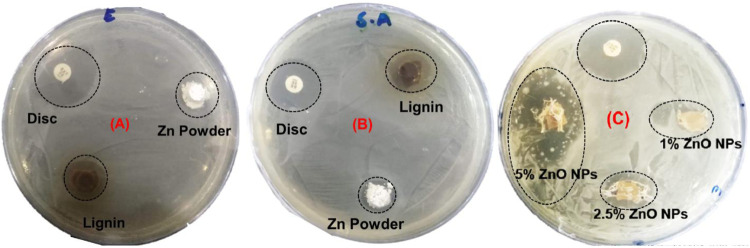
Zone of inhibition of synthesized Lignin@ZnO and commercial zinc oxide powder for **(A)** *E*. *coli*, **(B)** *S*. *aureus*, and **(C)** zone of inhibition of GTR membrane against *E*. *coli*.

Among all metal nanoparticles, ZnO NPs are biocompatible and show non-toxicity with human cells. These features of ZnO NPs make them potent against many microorganisms (Abbasi et al., 2017). The agar disk diffusion process was utilized to detect the antibacterial activity, as shown in Figure 7. This study evaluated the antibacterial efficacy of synthesized ZnO NPs, commercial zinc oxide powder (ZnO), and fabricated GTR membranes toward two bacterial strains *Escherichia coli* and the *Staphylococcus aureus*. Sterilization of membranes, by irradiation, has been examined and analyzed. All the samples were sterilized using UV light, which successfully inactivates microorganisms. The optical images showed that synthesized Lignin@ZnO and fabricated membranes showed strong antibacterial results compared to commercial zinc oxide powder and lignin alone, as shown in Figure 7A–C.

The comparative results are also shown in Figure 7. The antibacterial action in the membrane and ZnO NPs is due to the production of reactive oxygen species (ROS) and metal ions (Zn^2+^) (Aadil et al., 2019). These bind to the membrane of the bacteria and hinder the protein transport channels that disturb the transport of materials within the membrane. These can also attach to the DNA and different enzymes alter the shape and structure of the enzymes and DNA and disturb their functioning (Aadil et al., 2019). The bactericidal property of zinc oxide nanoparticles may be recognized for their capability to interrelate with the cell membrane of numerous bacterial classes. Zinc powerfully combines with proteins and lipids, altering the osmotic equilibrium and enhancing the membrane permeability (Abdulkareem et al., 2015). Furthermore, ZnO NPs enhance the oxidative pressure inside the bacteria cell due to their capability to produce Zn^2+^ ions and the reactive oxygen species (ROs) inside the microbial cells, which can also prevent the development of the planktonic bacterial species (Padovani et al., 2015). ZnO NPs have selected poisonousness to bacterial species and have antibacterial properties against spores (microorganisms) that are temperature-resistant and pressure-resistant (Murfadunnisa et al., 2019). Some studies display a negative association between the size of the nanoparticles and cytotoxicity. However, there is no such relation in the literature about this activity for the zinc oxide NPs (Garcia et al., 2018). ZnO NPs are also biocompatible and biologically safe with distinctive structural, thermal, and electrical applications, changing particle morphology, size, orientation, and shape (Abbasi et al., 2017).

**FIGURE 8:**
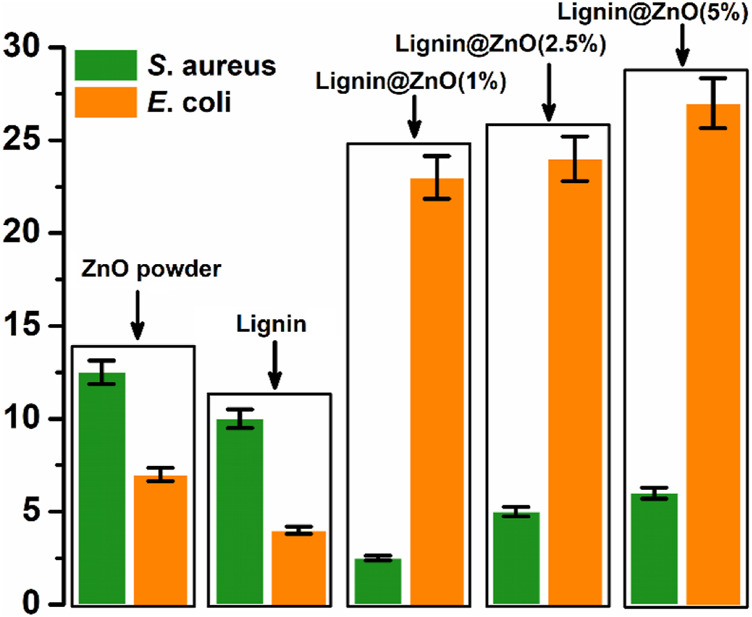
Zone of inhibition exhibited by the ZnO powder, Lignin, and 1, 2.5, and 5% Lignin@ZnO-GTR membrane against *S*. *aureus*, and *E. coli*.

The zone of inhibition around the membrane displays the limit of progress of the bacteria around the membrane locality. This procedure shows that the membrane and Lignin@ZnO NPs can prevent the gain of the microbes, so in the periodontal tissue, it can inhibit the colonization of the bacteria. The zone of inhibition is explained *via* the graph in Figure 8. The antibacterial property of Lignin@ZnO and membranes is greater than that of commercial ZnO powder. Lignin@ZnO showed greater efficacy for *S. aureus* than for *E. coli*. All membranes (1, 2.5, 5%) showed excellent antibacterial properties against *E*. *coli* compared to *S*. *aureus*. The inhibition zone test demonstrates that Lignin@ZnO-GTR has a better antimicrobial ability than free ZnO.

**FIGURE 9:**
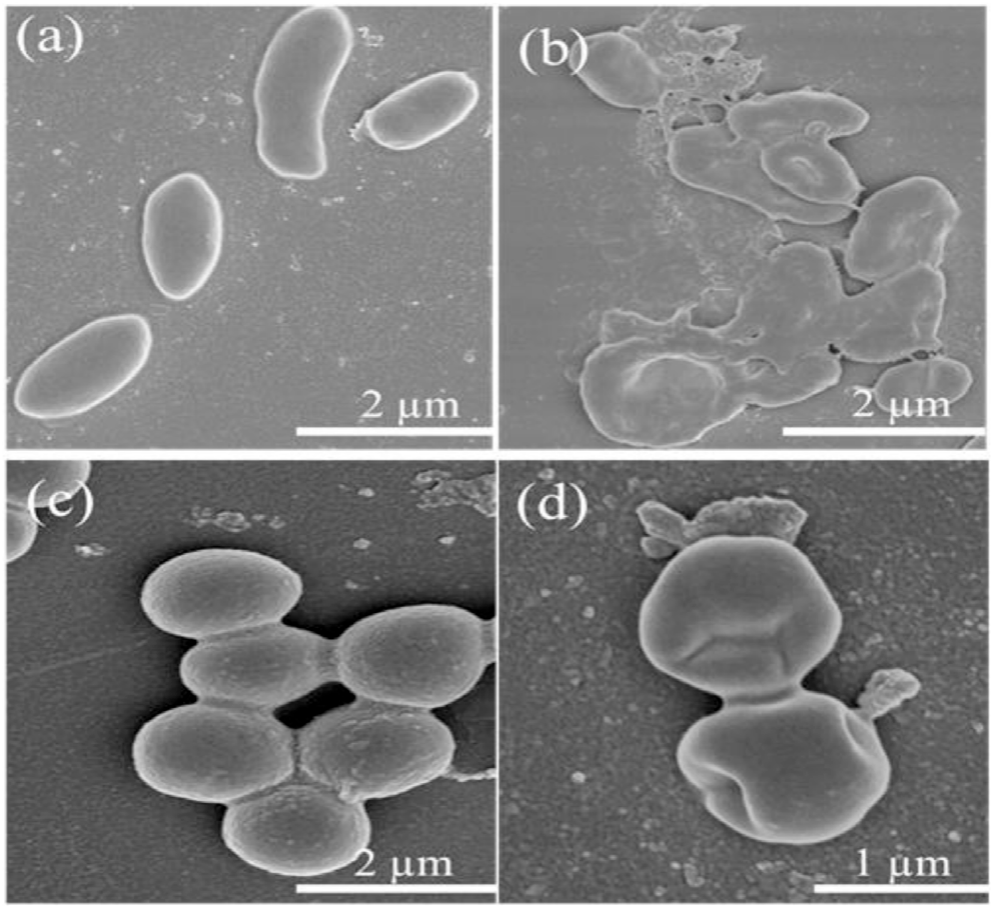
FESEM images of **(A–B)** *E. coli* and **(C–D)** *S. aureus* treated with different time: **(A,C)** 0 h and **(B,D)** 24 h.

Furthermore, the morphological changes of Lignin@ZnO-GTR toward the bacteria (*E. coli* and *S. aureus*) were observed using the field emission scanning electron microscope (FE-SEM). Intact *E. coli* and *S. aureus* in Figures 9A,C have distinct outer membranes, indicating that high-vacuum and high-energy electron beams do not affect bacteria cell structure. However, after co-incubation with Lignin@ZnO-GTR for 24 h, the cellular cohesion was weakened with heavy damage to the outer membranes (Figures 9B,D), and death of bacteria may occur due to cytoplasm outflow. In general, the cell of *E. coli* is less thick than *S. aureus*, and more damage to *E. coli* was observed (Abdul et al., 2019; Rauf et al., 2019; Liu et al., 2021).

## Conclusion

The present study fabricated the guided tissue regeneration membrane (GTR) using Lignin@ZnO and extracted mucilage from chia seeds. In periodontal regeneration, GTR membranes are seen as an attractive strategy. The addition of Lignin@ZnO nanoparticles to the membrane enhanced the antibacterial properties and strength of the membrane. The chemical structures and properties of the materials were characterized using UV–visible spectroscopy, FTIR, and SEM after every reaction process. Moreover, the FTIR results presented satisfactory relations between ZnO nanoparticles, polymer, and mucilage. The SEM images showed that the ZnO NPs have agglomeration and a diameter of less than 50 nm. The study of swelling and mechanical properties of fabricated membranes presented satisfactory results; degradation of the fabricated membrane was started after 24 h. The zone of inhibition against bacterial species for the zinc oxide nanoparticles and membrane was measured more than that for commercial zinc oxide powder. The antibacterial properties of Lignin@ZnO-based GTR membranes resulted in a reduction of *S. aureus* and *E*. *coli* viability. The membrane exhibited successful results, which proved the potential of the membrane to be used in periodontal regeneration. MTT biocompatibility and hemocompatibility assay can be performed to further evaluate the GTR membrane *in vivo* in a mouse model.

## Data Availability

The original contributions presented in the study are included in the article/Supplementary Material, further inquiries can be directed to the corresponding authors.
