# Supplementary material for: Fabrication of Guided Tissue Regeneration Membrane Using Lignin-Mediated ZnO Nanoparticles in Biopolymer Matrix for Antimicrobial Activity
Source: Front Chem. 2022 Apr 19;10:837858. doi: 10.3389/fchem.2022.837858 (PMC9063929; doi:10.3389/fchem.2022.837858)
Supplement: Supplementary file 1 [file DataSheet1.docx]

Fabrication of Guided Tissue Regeneration Membrane Using Lignin Mediated ZnO Nanoparticles in Biopolymer Matrix for Antimicrobial Activity

Bushra Bilal^a‡^, Rimsha Niazi^b‡^, Sohail Nadeem^b^, Muhammad Asim Farid^c^, Muhammad Shahid Nazir^a^, Toheed Akhtar^b^, Mohsin Javaid^b^, Aysha Mohyuddin^b^, Zulfiqar Ali^d^, Syed Ali Raza Naqvi^e^*, Nawshad Muhammad^f*^, Sadaf Ul Hassan^ab^*

Bushra Bilal^a‡^, Rimsha Niazi^b‡^, Sohail Nadeem^b^, Muhammad Asim Farid^c^, Muhammad Shahid Nazir^a^, Toheed Akhtar^b^, Mohsin Javaid^b^, Aysha Mohyuddin^b^, Zulfiqar Ali^d^, Syed Ali Raza Naqvi^e^*, Nawshad Muhammad^f*^, Sadaf Ul Hassan^ab^*, Eslam B. Elkaeed^g^, Hala A. Ibrahium^h, i^ Nasser S. Awwad^j^

^a^Department of Chemistry, COMSATS University Islamabad, Lahore Campus, 54000, Pakistan.

^b^Department of Chemistry, School of Sciences, University of Management and Technology, Lahore Campus, Pakistan.

^c^Department of Chemistry, Division of Science and Technology, University of Education, Vehari campuse, Pakistan.

^d^Department of Chemical Engineering, COMSATS University Islamabad, Lahore Campus, 54000, Pakistan.

^e^Department of Chemistry, Government College University, Faisalabad, Pakistan.

^f^Department of Dental Materials, Institute of Basic Medical Sciences, Khyber Medical University, Peshawar, Pakistan

^g^Department of Pharmaceutical Sciences, College of Pharmacy, Almaarefa University, Ad Diriyah 13713, Riyadh, Saudi Arabia.

^h^Biology Department, Faculty of Science, King Khalid University, P.O. Box 9004, Abha 61413, Saudi Arabia.

^i^Department of Semi Pilot Plant, Nuclear Materials Authority, P.O. Box 530, El Maadi, Egypt.

^j^Chemistry Department, Faculty of Science, King Khalid University, P.O. Box 9004, Abha 61413, Saudi Arabia.

*Corresponding Authors: [drarnaqvi@gmail.com](mailto:drarnaqvi@gmail.com) (Syed Ali Raza Naqvi); [sadaf.hassan@umt.edu.pk](mailto:sadaf.hassan@umt.edu.pk) (Sadaf Ul Hassan); [nawshad.ibms@kmu.edu.pk](mailto:nawshad.ibms@kmu.edu.pk) (Nawshad Muhammad)


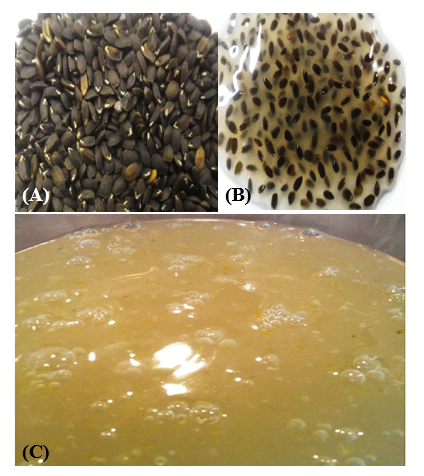


**Figure S1.** Chia seeds (A) before swelling in water (B) swelling in water (C) after extraction.


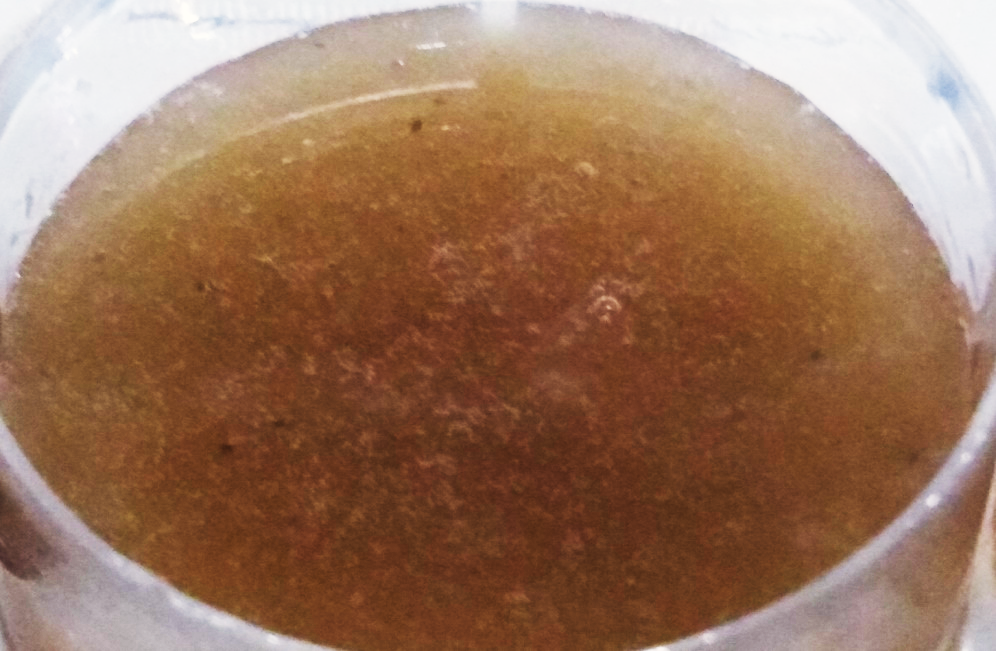


**Figure S2.** Membrane before drying.


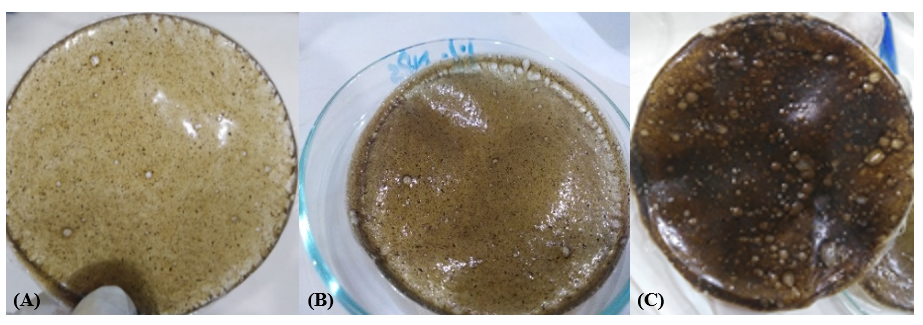


**Figure S3.** Prepared membranes after drying (A) membrane having 1% of lignin mediated zinc oxide nanoparticles (B) membrane having 2.5% nanoparticles (C) membrane having 5% of lignin mediated zinc oxide nanoparticles


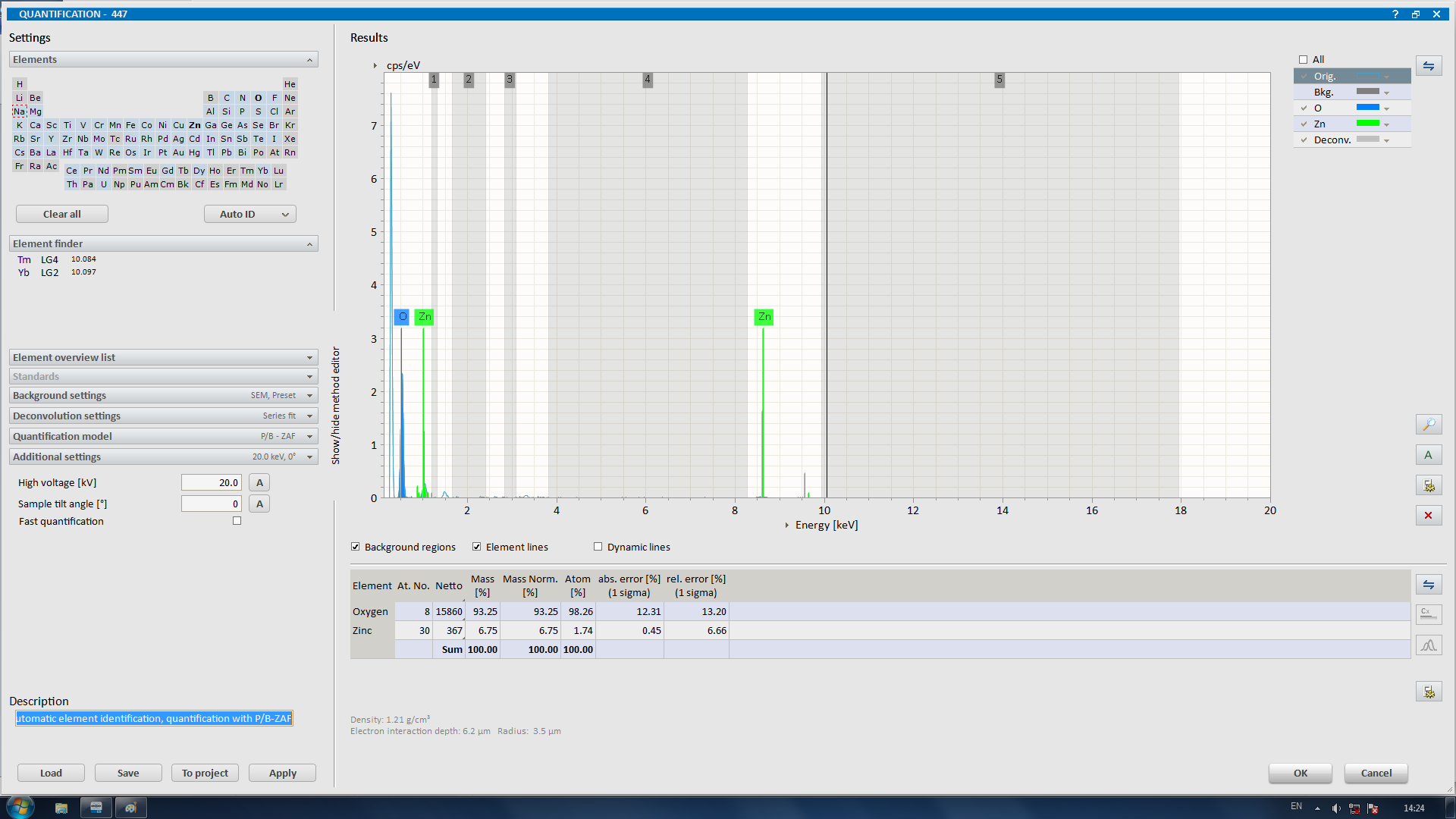


**Figure S4.** EDX of ZnO NP.


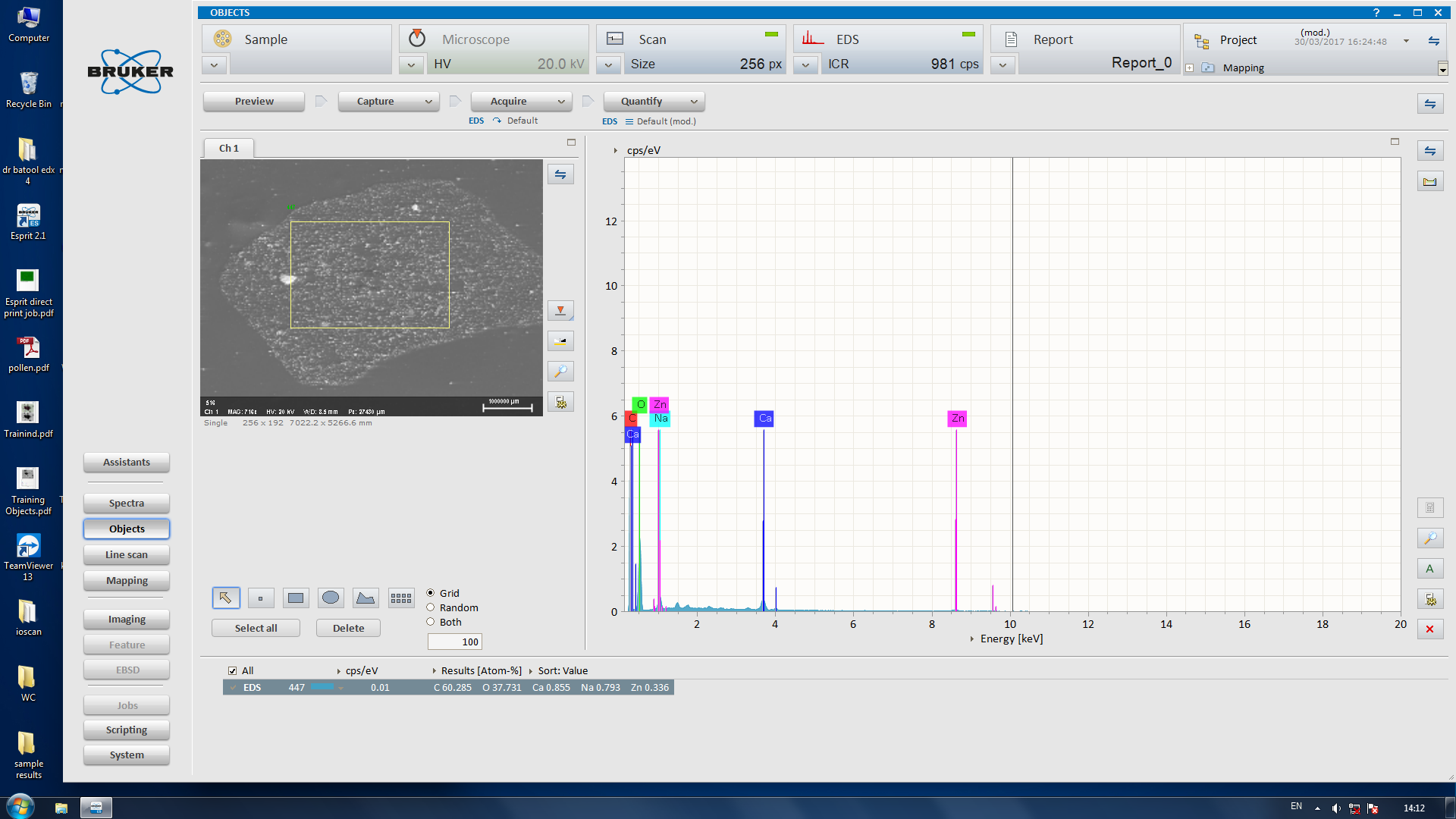


**Figure S5.** EDX of 1% lignin mediated ZnO NPs GTR membrane.
